# Impact of Experiencing Acute Coronary Syndrome Prior to Open Heart Surgery on Psychiatric Status

**DOI:** 10.5935/1678-9741.20160064

**Published:** 2016

**Authors:** Volkan Yüksel, Yasemin Gorgulu, Rugul Kose Cinar, Serhat Huseyin, Mehmet Bulent Sonmez, Suat Canbaz

**Affiliations:** 1 Trakya Üniversitesi Rektörlüğü, Balkan Yerleşkesi, Turkey.

**Keywords:** Acute Coronary Syndrome, Coronary Artery Bypass, Depression, Anxiety

## Abstract

**Objective:**

The incidence of depression and anxiety is higher in patients with acute coronary syndrome. The aim of this study is to determine whether experiencing acute coronary syndrome prior to open heart surgery affects patients in terms of depression, hopelessness, anxiety, fear of death and quality of life.

**Methods:**

The study included 63 patients who underwent coronary bypass surgery between January 2015 and January 2016. The patients were divided into two groups: those diagnosed after acute coronary syndrome (Group 1) and those diagnosed without acute coronary syndrome (Group 2). Beck depression scale, Beck hopelessness scale, Templer death anxiety scale and death depression scale, State-Trait anxiety inventory and WHOQOL-Bref quality of life scale were applied.

**Results:**

There was no significant difference between the two groups in terms of the total score obtained from Beck depression scale, Beck hopelessness scale - future-related emotions, loss of motivation, future-related expectations subgroups, death anxiety scale, the death depression scale, State-Trait Anxiety Inventory - social and environmental subgroups. The mental quality of life sub-scores of group 2 were significantly higher. The patients in both groups were found to be depressed and hopeless about the future. Anxiety levels were found to be significantly higher in all of the patients in both groups.

**Conclusion:**

Acute coronary syndrome before coronary artery bypass surgery impairs more the quality of life in mental terms. But unexpectedly there are no differences in terms of depression, hopelessness, anxiety and fear of death.

**Table:** 

Abbreviations, acronyms & symbols	
ACS	=Acute coronary syndrome
BDS	=Beck Depression Scale
CABG	=Coronary artery bypass surgery
CAD	=Coronary artery disease
COPD	=Chronic obstructive pulmonary disease
EF	=Ejection fraction
IHD	=Ischemic heart disease
LMCA	=Left main coronary artery disease
PAD	=Peripheral arterial disease
QOL	=Quality of life
STAI-S	=State of Anxiety Scale

## INTRODUCTION

Ischemic heart disease occurs as a result of the stenosis or occlusion of the coronary artery supplying the myocardium generally due to atherosclerosis. In most patients, the first symptom of ischemic heart disease (IHD) is acute coronary syndrome (ACS). Its typical findings are pressure sensation in the forefront of the chest and the compressing-type pain creeping the neck, jaw, shoulder and arm. Chronic ischemic coronary artery disease may progress asymptomatic in some patients and such patients are diagnosed with coronary artery disease (CAD) through examination made upon any suspicion^[1]^.

The incidence of depression and anxiety in patients with ACS is reported to be higher. Furthermore, fear of death, death-related depressive mood and accordingly a decline in quality of life (QOL) are observed in these patients^[2]^. Likewise, coronary artery bypass surgery (CABG) requirement also constitutes a factor leading to depression, anxiety, development of fear of death and decreased QOL^[3]^. CABG is both physical and psychological stressor. Patients consider this surgery as a life-threatening condition and a source of stress which will significantly impair their normal daily life pattern^[4]^. This stress may lead to depression, anxiety and fear of death, and impairs the QOL. In addition to some patients experiencing the stress caused by both ACS and CABG, there are some other who do not have ACS and ACS-related stress, but suffering only from the stress of CABG.

There is substantial evidence that depression is associated with morbidity and mortality in cardiac patients^[5]^. It is reported that the prevalence of depression before CABG is in the range of 20-47%^[6]^.

During the preparation for the surgery following the decision to proceed with bypass surgery; Beck depression scale (BDS), Beck hopelessness scale, Templer death anxiety scale and death depression scale, State-Trait anxiety inventory and WHOQOL-Bref QOL scale were performed. These scales applied in the study were validated and translated into Turkish language. Turkish language is the local language of the study group.

BDS is among the most commonly-used scales in mental health screening and researches on depression. A high total score indicates a high level or severity of depression. The BDS scores of 17 and above would identify a depression severe enough to require treatment with 90% accuracy^[7]^.

Templer's Death Anxiety Scale aimed at determining the level of death anxiety including a total of 15 items. Modelling some studies in the literature on his dissertation, it was transformed into a seven-point Likert scale and adapted to our population with the aim of a more robust measurement. Death Depression Scale was developed in 1990 by Templer. It aims to measure the mood states such as depression, sadness, loneliness, fear and grief experienced in relation to death. After its implementation, at least 0 and at most 17 points can be obtained. A score between 0 and 8 indicates no depressive mood whereas a score between 9 and 17 indicates a depressive mood - the higher the score, the more severe the depression^[8]^.

State-Trait Anxiety Inventory (STAI)-I, STAI-II scale was developed by Spielberger^[9]^. It consisted of State Anxiety Inventory and the Trait Anxiety Inventory, each with 20 items. The scores range from 20 (low anxiety) to 80 (high anxiety). It indicates no anxiety between points 36 and below, a slight anxiety between 37-42 and high anxiety between 43 and above. Overall, a high state and trait anxiety score shows a high level of anxiety, and it is stated that the individuals with scores above 60 need a professional help.

WHOQOL-Bref Quality of Life Scale was developed by the World Health Organization and consists of 26 items through which the overall perceived QOL is questioned. The study includes the sections of physical health, mental health, social health and environmental health. Physical health section includes the questions designated for investigating ability to perform everyday tasks, dependence on medication and treatment, vitality and fatigue, mobility, pain and discomfort, sleep and rest and ability to work whereas mental health section includes questions related to positive and negative emotions, self-esteem, body image and appearance, personal beliefs and attention, and social section includes questions related to relationships with other people, social support and sexual life, and finally, environment and national environmental section includes questions related to home environment, physical security and safety, financial resources, opportunity to receive health services, physical environment and transport. The higher the score obtained in the scale, the higher the QOL.

It was observed that, in terms of depression, anxiety and fear of death, there are some differences between the patients with ACS for whom a bypass surgery is projected and those who were diagnosed with IHD in consequence of their consultation due to other medical reasons and for whom a bypass surgery is projected. We hypothesized that experiencing ACS prior to open heart surgery may affect the psychiatric status of those patients. The aim of this study is to determine whether there is a statistically significant difference between these two groups in terms of depression, anxiety, level of hope for the future, fear of death and QOL. To our knowledge, our study is the first study in the literature on this subject.

## METHODS

The study population included 63 patients who underwent a primary CABG under elective conditions between January 2015 and January 2016 in the cardiovascular surgery clinic. The patients were divided into two groups: those diagnosed after experiencing an ACS (Group 1) and those diagnosed without an ACS (Group 2). Group 1 consisted of the patients for whom a bypass surgery is to be performed after an ACS. Group 2 was, however, consisted of the patients for whom a bypass surgery is decided to be performed with the diagnosis of ischemic heart disease as a result of examinations made upon patients' consultation to the hospital due to other medical reasons upon suspicion.

As a result of interviews conducted with patients, those with previous psychiatric symptoms or treatment history, those who had previously undergone a heart surgery and those for whom an emergency surgery is planned were excluded. We excluded the patients who have previous psychiatric history because of this history could confound the psychiatric states of patients and for most truly examination of the only CABG's effect on psychiatric statement.

In addition, the personal information form created by researchers and containing data such as age, gender, diabetes, hypertension, hyperlipidemia, chronic obstructive pulmonary disease (COPD), smoking, peripheral arterial disease (PAD), left main coronary artery disease (LMCA), ejection fraction (EF) and the number of vessels bypassed was completed for each patient.

BDS is among the most commonly-used scales in mental health screening and researches on depression. A high total score indicates a high level or severity of depression. In her validity and reliability study, Hisli^[10]^ determined the cut-off as 17 points, and reported that the BDS scores of 17 and above would identify a depression severe enough to require treatment with 90% accuracy.

Beck Hopelessness Scale aimed at determining the level of pessimism about the future of an individual. The high scores show that the hopelessness of the individual is high. The scale is composed of three factors including "Future-Related Emotions", "Loss of Motivation" and "Future-Related Expectations". Beck et al.^[7]^ classified the subjects in four groups according to their responses and reported no hopelessness in 0 to 3, slight hopelessness in 4 to 8, moderate hopelessness in 9 to 14 and severe hopelessness in 15 to 20. The validity and reliability study was performed by Durak & Palabıyıkoğlu^[11]^, in 1994. The high scores show that the hopelessness of the individual is high. The scale is composed of three factors, including "Future-Related Emotions", "Loss of Motivation" and "Future-Related Expectations".

Templer's Death Anxiety Scale aimed at determining the level of death anxiety, including a total of 15 items. Modelling some studies in the literature on his dissertation, Ertufan^[12]^ transformed it into a seven-point Likert scale and adapted to Turkish with the aim of to become a more robust measurement.

Death Depression Scale was developed in 1990 by Templer and the study of validity and reliability of the scale in Turkey was performed by Yaparel & Yıldız^[13]^. It aims to measure the mood states such as depression, sadness, loneliness, fear and grief experienced in relation to death. After its implementation, at least 0 and at most 17 points can be obtained. A score between 0 and 8 indicates no depressive mood whereas a score between 9 and 17 indicates a depressive mood - the higher the score, the more severe the depression^[14]^.

STAI-I, STAI-II scale was developed by Spielberger^[9]^. It consisted of State Anxiety Inventory and the Trait Anxiety Inventory, each with 20 items. Öner & LeCompte translated the scale into Turkish and performed its validity and reliability study^[15]^.

WHOQOL-Bref Quality of Life Scale was developed by the World Health Organization and consists of 26 items through which the overall perceived QOL is questioned. Eser et al.^[16]^ performed the study of validity and reliability of the scale in Turkey. The higher the score obtained in the scale, the higher the QOL.

### Statistical Analysis

The data were expressed as the mean ± standard deviation. Intergroup comparison was performed with the Mann-Whitney U-test. Comparisons of categorical variables were made using a chi-square test. Student's t-test was used for comparison of continuous data. Spearman correlation analysis was used to measure the strength of association between variables. The data were examined using SPSS 20 for Windows (SPSS Inc., Chicago, IL, USA). Statistical differences were considered significant if *P*<0.05.

Ethical committee approval for the study was obtained from Non-Invasive Clinical Research Ethics Committee (096/2014). Guidelines on patient consent have been met and any details of informed consent were obtained.

## RESULTS

The demographic variables were compared in Table 1. There was no significant difference in gender distribution and mean age of the groups. Except for hyperlipidemia, no significant difference was found between the groups in comparison of the preoperative demographic data with operative data. Hyperlipidemia was significantly higher in Group 2 (*P*<0.05).

**Table 1:** Preoperative and operative patient characteristics and data.

	Group 1 Number of patients (%)	Group 2 Number of patients (%)
Age	61±9.2	62.9±10.2
Gender (male/female)	22/8	28/5
Hypertension	23 (76.7)	28 (84.8)
Hyperlipidemia	6 (20)	17 (51.5)
Smoking	18 (60)	18 (54.5)
Chronic obstructive pulmonary disease	1 (3.3)	5 (15.2)
Peripheral arterial disease	0	1 (3)
Left main coronary artery disease	4 (13.3)	4 (12.1)
Ejection fraction	58±7.3	57.3±7.4
Diabetes mellitus	9 (30)	14 (42.4)
Number of grafts	2.6±0.7	2.7±0.8

No significant difference was found between the two groups in terms of the total score obtained from BDS, Beck hopelessness scale - future-related emotions, loss of motivation, future-related expectations subgroups, death anxiety scale, the death depression scale, State-Trait Anxiety Inventory - social and environmental subgroups. During the evaluation of WHOQOL-Bref mental subgroup, the mental QOL sub-scores of group 2 were significantly higher than those with ACS (*P*=0.018). Patients' assessments based on the scales are shown in detail in the Tables 2 and 3. There was no significant difference between the two groups in terms of patients' scores of death anxiety scale (*P*=0.75). However, when all patients were considered together, it was found that 51.6% of patients had moderate anxiety, 24.2% had severe anxiety and 24.2% had slight anxiety. Again, there was no significant difference (*P*=0.90) between the two groups in terms of the scores obtained in the death depression scale and when considered in whole, a death-related depressive mood was observed in 72.6% of patients. In the group of patients with ACS, however, a significant positive correlation was found between BDS and Templer's death anxiety scale (r=0.382, *P*<0.05). A significant positive correlation was also found between Templer's death anxiety scale and death depression scale in the groups of patients both with ACS (r=0.760, *P*<0.001) and without ACS (r=0.502, *P*<0.05).

**Table 2:** Patients’ scale scores.

	BDS	BHS-em	BHS-mot	BHS-exp	BHS total	DAS	DDS
Group 1	14.9±9.5	2.2±0.9	3.0±2.1	5.8±1.3	11.1± 2.3	7.1±3.2	11.0±3.8
Group 2	12.1±7.4	2.1±0.6	3.1±2.0	5.6±1.4	10.8±1.8	6.7±3.1	9.4±3.3
*P*	0.36	0.68	0.86	0.60	0.51	0.59	0.12

BDS=Beck Depression scale; BHS=Beck hopelessness scale; em=future-related emotions; mot=loss of motivation, exp=futurerelated expectations; DAS=Templer’s death anxiety scale; DDS=death depression scale

**Table 3:** Patients’ scale scores.

	STAI-I	STAI-II	WHOQOL mh	WHOQOL ph	WHOQOL sh	WHOQOL eh	WHOQOL total
Group 1	51.4±5.2	48.3±7.0	23.7±4.2	21.8±2.8	10.1±2.5	27.1±4.5	82.8±11.1
Group 2	48.3±10.4	47.7±9.3	21.0±3.5	22.2±2.7	11.0±2.0	28.7±3.6	81.6±11.3
*P*	0.08	0.97	< 0.05*	0.50	0.24	0.21	0.80

STAI-I-II=state-trait anxiety inventory I-II; ph=physical health, mh=mental health; sh=social health; eh=environmental health

Although no significant difference was found between the two groups, the patients who are prepared for a bypass surgery, regardless of whether or not they are diagnosed with ACS, were found to be depressed and hopeless about the future and their levels of anxiety were found to be high.

Although no significant difference was found between the two groups in terms of the means of total score's obtained from BDS; we assessed the all patient's depressive levels according to the BDS points. According to the BDS scores; 28 of 63 patients obtained a score 17 and above while 14 patients scored between 12 and 17. So, 42 of 63 patients have major or minor levels of depressive symptoms. These scores suggest that the majority of the patients have depressive symptoms. According to the STAI's scores, anxiety levels were found to be significantly higher in all of the patients in both groups.

## DISCUSSION

Most of the patients hospitalized for CABG experience various emotions ranging from a slight fear to anxiety, depression and fear of death^[17]^. According to Spielberger^[9]^, anxiety is an unpleasant emotional state or condition which is characterized by subjective feelings of tension, apprehension and worry and by activation or arousal of the autonomic nervous system. Koivula et al.^[2]^ stated that waiting for CABG makes many patients more disturbed than their chest pain. Arthur et al.^[18]^ described that long wait for CABG can result in deterioration of patient's emotional state and physical activity. Moderate anxiety before major surgery is a normal emotion as it prepares the organism for the coming stress situation^[19]^. However, intense fear and anxiety are especially detrimental to cardiac patients because of the activation of sympathetic and parasympathetic nervous systems and cardiovascular excitation^[20]^.

Psychological problems such as depression and anxiety are widely reported soon after CABG surgery and remain evident for around one-fifth of patients one year after surgery^[21,22]^. There are many studies assessing patients' level of depression and anxiety^[23]^. However, the number of studies in which patients' level of preoperative depression, hopelessness and deathrelated anxiety as well as their relation to the QOL are assessed, are relatively limited. In our study, the scores from death anxiety scale of both groups are high. The idea of undergoing a CABG increased patient death anxiety independent of whether they have ACS previously or not.

Patients in both groups have received high scores from death depression scale, in a level indicating the presence of a depressive mood. Although the total DDS score was found to be higher in the first group compared to that of the second group, there was no significant difference between the two groups. Being about to undergo a bypass surgery both increase patients' death anxiety and creates a depressed mood related to the death.

Measured on the Spielberger State of Anxiety Scale (STAI-S) the prevalence of anxiety ranged between 22% and 48% in patients with CAD, prior to heart surgery^[24]^. Different studies show a moderate degree of anxiety (score on STAI-S 35.6-49.8) after myocardial infarction, before heart surgery^[24,25]^. In the study that evaluates the anxiety level of 190 patients one day before the CABG, the STAI mean score was found to be 35.8±10.4^[26]^. State and trait anxiety scores of our patients were found to be higher compared to this study.

It is known, patients experiencing level of preoperative distress were more likely to report decrements in several domains of QOL after their operation^[27]^. Better QOL was associated with lower anxiety level^[28]^. An increasing body of research evidence shows that the waiting period is difficult for patients. It has been found that waiting for surgery impairs the QOL for patients on the waiting list^[29]^. It has been found that during the waiting period to CABG patients suffer from impaired functional status, anxiety, depression and fear of death, and that the situation also adversely affects the patient's family and social relations^[30]^. It was also determined in our study that the QOL of the patients with ACS was mentally significantly impaired compared to those who do not have ACS.

The sample size in our study constitutes a limitation for our study. However, this is the first study to explore whether there is a difference between the patients with ACS and those without ACS, both prepared to a CABG, in terms of depression, anxiety, and the level of hope, death-related anxiety and QOL.

## CONCLUSION

In conclusion, depression, anxiety, hopelessness, deathrelated depression and anxiety level of the patients who will undergo an open heart surgery is high and the QOL in these patients is affected by this situation when both groups are considered together. But unexpectedly there are no differences in terms of depression, hopelessness, anxiety and fear of death. Also ACS before CABG impairs more the QOL in mental terms compared to those without ACS.

**Table:** 

Authors’ roles & responsibilities	
VY	Conception and study design; analysis and/or data interpretation; manuscript writing or critical review of its content; final manuscript approval
YG	Conception and study design; realization of operations and/ or trials; analysis and/or data interpretation; statistical analysis; final manuscript approval
RKC	Conception and study design; realization of operations and/ or trials; manuscript writing or critical review of its content; statistical analysis; final manuscript approval
SH	Conception and design study; analysis and/or data interpretation; final manuscript approval
MBS	Realization of operations and/or trials; manuscript writing or critical review of its content; final manuscript approval
SC	Conception and study design; realization of operations and/or trials; analysis and/or data interpretation; manuscript writing or critical review of its content; final manuscript approval
